# Association of Pathologic Response and Adjuvant Chemotherapy with Survival in Resected Pancreatic Ductal Adenocarcinoma Following Neoadjuvant Therapy

**DOI:** 10.3390/cancers17111797

**Published:** 2025-05-28

**Authors:** James Yu, Jose M. Laborde, Robin Park, Moazzam Shahzad, Youngchul Kim, Jaekyung Cheon, Iman Imanirad, Richard D. Kim, Tiago Biachi de Castria, Nicole L. Nardella, Mokenge Malafa, Jason W. Denbo, Jason B. Fleming, Sarah E. Hoffe, Jessica M. Frakes, Andrew J. Sinnamon, Jose M. Pimiento, Pamela J. Hodul, Dae Won Kim

**Affiliations:** 1 Division of Hematology and Medical Oncology, H. Lee Moffitt Cancer Center and Research Institute, University of South Florida, Tampa, FL 33612, USA; james.yu@moffitt.org (J.Y.); robin.park@moffitt.org (R.P.); moazzam.shahzad@moffitt.org (M.S.); 2 Department of Biostatistics and Bioinformatics, H. Lee Moffitt Cancer Center and Research Institute, Tampa, FL 33612, USA; jose.laborde@moffitt.org (J.M.L.); youngchul.kim@moffitt.org (Y.K.); 3 Department of Gastrointestinal Oncology, H. Lee Moffitt Cancer Center and Research Institute, Tampa, FL 33612, USA; jaekyung.cheon@moffitt.org (J.C.); iman.imanirad@moffitt.org (I.I.); richard.kim@moffitt.org (R.D.K.); tiago.biachi@moffitt.org (T.B.d.C.); nicole.nardella@moffitt.org (N.L.N.); mokenge.malafa@moffitt.org (M.M.); jason.denbo@moffitt.org (J.W.D.); sarah.hoffe@moffitt.org (S.E.H.); jessica.frakes@moffitt.org (J.M.F.); andrew.sinnamon@moffitt.org (A.J.S.); jose.pimiento@moffitt.org (J.M.P.); pamela.hodul@moffitt.org (P.J.H.); 4 Department of Surgery, UT Southwestern Medical Center, Dallas, TX 75390, USA; jason.fleming@utsouthwestern.edu

**Keywords:** pancreatic cancer, adjuvant chemotherapy, neoadjuvant therapy, pathologic response

## Abstract

**Background:** In patients with curatively resected pancreatic adenocarcinoma who have undergone neoadjuvant chemotherapy (NACT), evidence supporting the benefit of additional adjuvant chemotherapy (ACT) remains limited. We aim to identify favorable factors contributing to survival benefits in resected pancreatic adenocarcinoma after NACT. **Methods**: This is a retrospective cohort study of pancreatic adenocarcinoma patients who underwent NACT followed by curative surgical resection between 2008 and 2023 at a single academic institution. Univariate and multivariable analyses were conducted to identify factors contributing to disease-free survival (DFS) and overall survival (OS). **Results**: A total of 230 patients with a median age of 68 years (IQR, 62–72 years) were included. All patients underwent curative surgical resection. Of these, 42% received neoadjuvant modified (m) FOLFIRINOX (96/230), 15% received gemcitabine plus nab-paclitaxel (GEM-NAB) (34/230), and 43% received gemcitabine, docetaxel, and capecitabine (GTX) (100/230). In univariate analysis, lower College of American Pathologists (CAP) tumor regression grade (TRG) (0–1 vs. 2–3, median DFS: 29.8 vs. 14.2 months, *p* = 0.0081) and receipt of ACT (Yes vs. No, median DFS: 22.2 vs. 12.4 months, *p* < 0.0001) demonstrated significant associations with superior DFS. Multivariable analysis identified receipt of ACT as an independent predictor of superior DFS (HR 0.55, 95% CI: 0.39–0.78, *p* = 0.0007) and OS (HR 0.49, 95% CI: 0.33–0.71, *p* = 0.0002). However, the NACT regimen (mFOLFIRINOX vs. GEM-NAB) and the transition between neoadjuvant and adjuvant therapy (de-escalation vs. continuation vs. change) did not correlate with DFS or OS. The duration of perioperative chemotherapy showed a trend toward improved survival outcomes, though not statistically significant (6 months vs. <6 months: DFS, 19.4 vs. 16.2 months, *p* = 0.1448; OS, 49.6 vs. 30.4 months, *p* = 0.0623). In the following subgroup analyses, receipt of ACT provided DFS/OS benefits in patients who did not achieve a major pathologic response, pN0, or R0 resection (DFS: *p* = 0.0003; OS: *p* < 0.0001). However, it did not provide DFS/OS benefits in those who achieved a major pathologic response with pN0/R0 to NACT (DFS: *p* = 0.8036; OS: *p* = 0.1877). **Conclusions**: In resected pancreatic adenocarcinoma following NACT, receiving ACT was associated with favorable survival outcomes. Additional ACT appears to benefit patients who did not achieve a major pathologic response (pN0 or R0) to neoadjuvant therapy, with limited benefit for those who achieved a major response with pN0/R0. The specific NACT regimen (mFOLFIRINOX vs. GEM-NAB) and changes in ACT from NACT did not significantly influence survival outcomes in our cohort.

## Simple Summary

The optimal treatment strategy following neoadjuvant therapy and curative resection in early-stage pancreatic cancer remains inconclusive. We retrospectively evaluated the clinicopathologic features and survival outcomes of patients with curatively resected pancreatic cancer treated with neoadjuvant chemotherapy. Receiving adjuvant chemotherapy (ACT) was associated with favorable survival outcomes. Subgroup analyses showed that ACT appeared to benefit patients who did not achieve a major pathologic response, pN0, or R0 status following neoadjuvant therapy, whereas those who achieved a major response with pN0/R0 showed no survival benefit. The specific neoadjuvant chemotherapy regimen (FOLFIRINOX vs. GEM-NAB) and changes in ACT from NACT did not significantly influence survival outcomes in our cohort.

## 1. Introduction

In recent years, the global incidence of pancreatic cancer has been increasing, particularly among younger individuals; however, the overall 5-year relative survival rate remains poor, ranging from 7.2% to 14.3% [1,2,3]. Approximately only 15–20% of pancreatic cancer patients present with resectable disease [4,5,6]. Approximately half of resections for pancreatic cancer are microscopically incomplete (R1), placing patients at a high risk of recurrence [7,8,9]. An increasing number of cases are being treated with upfront neoadjuvant therapy (NACT) to facilitate downstaging, achieve microscopically negative margin (R0) resection, control early systemic spread, and ultimately improve survival outcomes in early-stage pancreatic cancer [10]. Currently, for borderline resectable and locally advanced pancreatic ductal adenocarcinoma (PDAC), NACT is considered the standard approach [11].

Despite the growing use of NACT in PDAC, very limited data are available to guide adjuvant chemotherapy (ACT) strategies following NACT and curative resection. Key clinical questions remain, including determining the optimal ACT regimen and identifying patients who derive genuine clinical benefit from additional adjuvant therapy following neoadjuvant therapy and surgical resection. Several retrospective studies have reported mixed results regarding the benefit of ACT in PDAC patients treated with upfront NACT, with some studies demonstrating benefit across the entire patient population, while others suggest that the benefit is primarily observed in subpopulations such as patients showing lymph-node-metastases [12,13,14].

In this study, we present the experience of a single academic institution in managing PDAC patients who underwent curative surgical resection following NACT. Our aim is to evaluate prognostic factors associated with survival outcomes and identify potential predictive factors that could optimize adjuvant therapy strategies for early-stage PDAC treated with curative resection after NACT.

## 2. Materials and Methods

### 2.1. Patients and Study Population

This nonrandomized, retrospective study was conducted at Moffitt Cancer Center and included patients treated between June 2008 and December 2023. Inclusion criteria comprised individuals with (1) histologically confirmed localized PDAC, (2) who underwent NACT, and (3) who underwent curative surgical resection. Patients with (1) other histologic types, (2) distant metastasis at the initial presentation, (3) upfront surgery without NACT, or (4) limited related data, such as the absence of a pathology report for surgical resection, were excluded. This study received approval from the relevant Ethical and Independent Institutional Review Board at Moffitt Cancer Center and adhered to the principles outlined in the Declaration of Helsinki.

### 2.2. Treatment Evaluation and Outcome Definitions

Patients in this study were treated with one of the following NACT regimens: (1) modified FOLFIRINOX (mFOLFIRINOX), (2) gemcitabine and nab-paclitaxel (GEM-NAB), or (3) gemcitabine, docetaxel, and capecitabine (GTX). Curative pancreatic cancer surgery was performed following NACT based on the decision of a multidisciplinary team. Tumor resectability was assessed according to the National Comprehensive Cancer Network (NCCN) criteria [11]. For the transition from neoadjuvant to adjuvant therapy, patients were categorized into three groups: (1) Same, where the ACT regimen was identical to the NACT regimen; (2) De-escalation, in which the ACT regimen consisted of fewer chemotherapy agents but included components of the NACT regimen; and (3) Conversion, where the ACT regimen was switched to a different regimen, such as gemcitabine-based chemotherapy from mFOLFIRINOX. The transition from NACT to ACT was based on the discretion of the treating physician without a pre-specified protocol.

Disease-free survival (DFS) was defined as the interval from the date of curative surgery to the date of recurrence, death from any cause, or the last follow-up date. Overall survival (OS) was defined as the period from the date of surgery to the date of death or the last follow-up. Patients who remained event-free at the time of the last follow-up were censored. The response to NACT was evaluated using the Tumor Regression Grade (TRG) system established by the College of American Pathologists (CAP), which employs a four-tier grading system: grade 0 (no viable cancer cells), grade 1 (single cells or rare small clusters of cancer cells), grade 2 (residual cancer with evident tumor regression but more than single cells or rare small clusters), and grade 3 (extensive residual cancer with no evidence of tumor regression) [15]. Various scoring systems exist for assessing tumor response following NACT in resected pancreatic cancer; however, a recent systematic review identified the CAP scoring system as having the lowest risk of bias [14,16]. Postoperative tumor staging was determined using the American Joint Committee on Cancer (AJCC) Eighth Edition guidelines [17]. The major pathological response (MPR) was defined as TRG-CAP 0-1 [18].

### 2.3. Statistical Analysis

Baseline patient characteristics were summarized as median values with interquartile range (IQR) for continuous variables and as frequencies and proportions (%) for categorical variables. The Kaplan–Meier method was used to estimate DFS and OS, with comparisons performed using the log-rank test. Both univariate and multivariable Cox proportional hazards regression analyses were performed to evaluate hazard ratios (HR) and corresponding 95% confidence intervals (CI) based on various candidate prognostic clinicopathologic factors. A two-sided *p*-value of ≤0.05 was considered statistically significant. All statistical analyses were conducted using R software, version 4.2.3.

## 3. Results

### 3.1. Baseline Characteristics

A total of 230 patients were included in the study, with a median follow-up of 21.4 months (range, 0.3–107.3 months). Table 1 summarizes the baseline characteristics of the cohort. The study population consisted of 51% males (117/230) and 49% females (113/230), with a median age of 68 years (IQR, 62–72 years). Patients received NACT regimens as follows: mFOLFIRINOX in 42% (96/230), GEM-NAB in 15% (34/230), and GTX in 43% (100/230). Only 3% (7/230) completed the 6-month-long NACT. Additionally, 67% (155/230) of patients received ACT following curative resection. MPR with pN0/R0 was achieved in 20% (46/230) of patients.

### 3.2. Prognostic Factors Associated with Survival Outcomes

**Figure 1:**
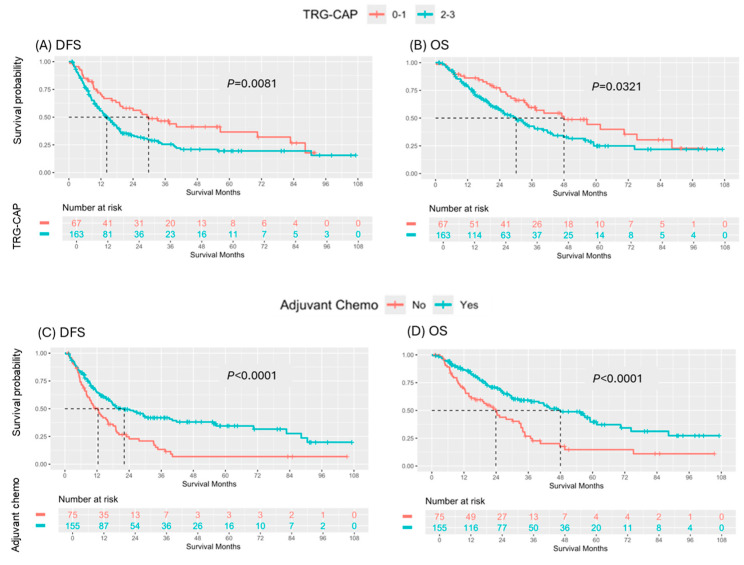
Comparison of survival outcomes by Tumor Regression Grade College of American Pathologists (TRG-CAP) score and Receipt of Adjuvant Chemotherapy: (**A**) DFS and (**B**) OS based on TRG-CAP, and (**C**) DFS and (**D**) OS based on receipt of adjuvant chemotherapy in patients with pancreatic ductal adenocarcinoma treated with curative resection following neoadjuvant therapy.

**Figure 2:**
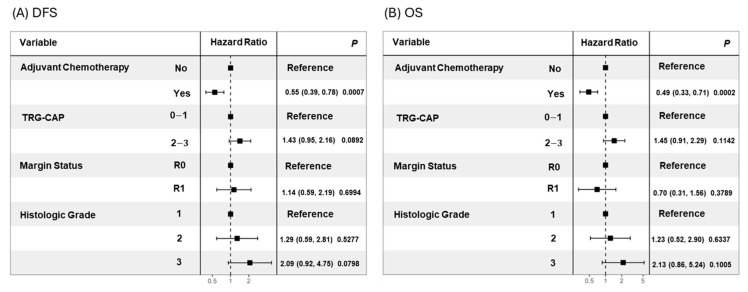
Forest plots for multivariable Cox regression of (**A**) DFS and (**B**) OS.

In the analysis of candidate prognostic factors, univariate analysis demonstrated that lower TRG-CAP (0–1 vs. 2–3; median DFS: 29.8 vs. 14.2 months, *p* = 0.0081) and receipt of ACT (Yes vs. No; median DFS: 22.2 vs. 12.4 months, *p* < 0.0001) were significantly associated with superior DFS (Figure 1). Both lower TRG (0–1 vs. 2–3; median OS: 48.0 vs. 30.1 months, *p* = 0.0321) and receipt of ACT (Yes vs. No; median OS: 48.0 vs. 23.9 months, *p* < 0.0001) were also significantly associated with increased OS (Figure 1). Multivariable analysis identified receipt of ACT as an independent predictor of superior DFS (hazard ratio [HR]: 0.55, 95% confidence interval [CI]: 0.39–0.78, *p* = 0.0007) and OS (hazard ratio [HR]: 0.49, 95% confidence interval [CI]: 0.33–0.71, *p* = 0.0002) (Figure 2).

The total duration of perioperative chemotherapy showed a trend toward improved survival outcomes, although statistical significance was not reached (6 months vs. <6 months: DFS, 19.4 vs. 16.2 months, *p* = 0.1448; OS, 49.6 vs. 30.4 months, *p* = 0.0623) (Supplement Figure S1). However, the NACT regimen (mFOLFIRINOX vs. GEM-NAB), NACT duration, and treatment transition from neoadjuvant to adjuvant therapy (de-escalation vs. continuation vs. conversion) were not significantly correlated with DFS or OS (Supplement Figure S2).

### 3.3. Subgroup Analyses by Pathologic Response

**Figure 3:**
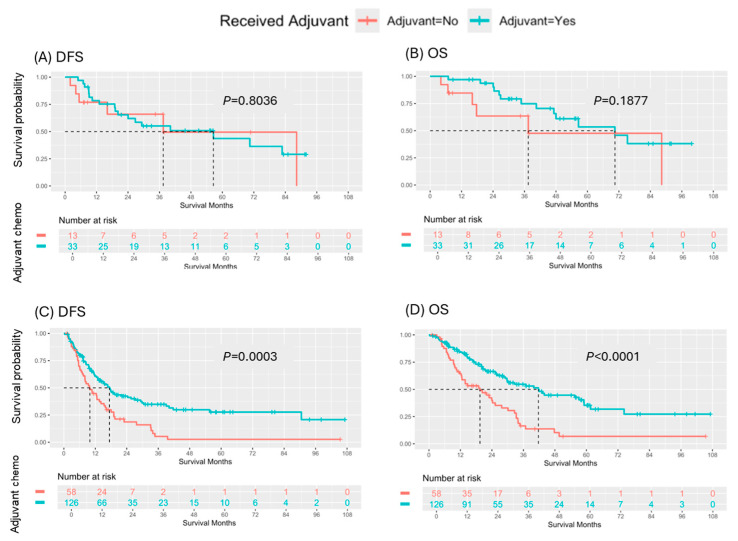
Survival outcomes by major pathologic response with pN0/R0. (**A**) DFS and (**B**) OS based on receipt of adjuvant chemotherapy in patients who achieved a MPR with pN0/R0 following neoadjuvant chemotherapy. (**C**) DFS and (**D**) OS by receipt of adjuvant chemotherapy in patients who did not achieve a MPR, pN0 or R0 from neoadjuvant therapy.

Among the 46 patients who achieved an MPR with N0 and R0 (TRG-CAP 0–1, pN0, R0 resection), receipt of ACT did not significantly impact DFS (*p* = 0.8036) or OS (*p* = 0.1877) (Figure 3). Conversely, among the 184 patients who did not achieve an MPR, pN0, or R0 resection, receipt of ACT was significantly associated with both DFS (median: 9.9 vs. 17.4 months, *p* = 0.0003) and OS (median: 19.4 vs. 41.7 months, *p* < 0.0001) (Figure 3).

Forty patients had TRG-CAP 3 with a median DFS of 15.4 months (IQR 8.4–29.3). Among them, thirty patients received ACT, including 14 who converted ACT from NACT, with a median DFS of 18.4 months (IQR 15.1–not reached); 14 who de-escalated ACT, with a median DFS of 15.1 months (IQR 10.6–40.2); and 2 who received the same ACT as NACT, with DFS of 16.2 and 9.8 months, respectively.

## 4. Discussion

The use of NACT has been increasing in early-stage pancreatic cancer, aimed at controlling potential early systemic spread, facilitating tumor downstaging to achieve R0 resection, and ultimately improving survival outcomes [10]. This approach is now considered the standard of care for borderline resectable and locally advanced PDAC and is also an option for patients with high-risk resectable disease [11]. Despite the growing adoption of NACT, very limited data are available to guide post-operative management in resected PDAC following NACT. Current guidelines, including those from the NCCN and the European Society for Medical Oncology (ESMO), do not provide specific recommendations regarding ACT in this setting [11,19]. The American Society of Clinical Oncology (ASCO) guidelines recommended completing a total of six months of perioperative chemotherapy, based primarily on extrapolated data from ACT trials [20]. Given the fact that recurrence rates remain high with poor long-term survival outcomes despite neoadjuvant therapy followed by resection, there is an unmet need to establish management for optimizing adjuvant treatment in this setting [10,21].

Analysis of our single-institution experience revealed that achieving TRG-CAP 0–1 from NACT, and receiving additional ACT were associated with improved survival outcomes. However, the type of NACT regimen, duration of NACT, and the transition from NACT to ACT did not demonstrate a significant correlation with survival outcomes. Subgroup analyses revealed that ACT provided a survival benefit in patients who did not achieve an MPR (TRG-CAP 0–1), pN0, or R0 resection but showed no benefit in those who achieved MPR with pN0/R0. This suggests that MPR with pN0/R0 may serve as a potential predictive factor to guide the decision on whether to administer additional ACT in patients undergoing NACT followed by resection in PDAC. In our cohort, 20% (46/230) of patients achieved MPR with pN0/R0 from NACT.

Various retrospective studies have evaluated the association between ACT and survival outcomes in PDAC patients who underwent curative resection following NACT. These studies have generally demonstrated that ACT is most likely to provide a survival benefit in patients with aggressive, residual disease and a suboptimal response to NACT [12,13,22]. Roessel et al. conducted a retrospective study evaluating the benefit of ACT in patients who received neoadjuvant FOLFIRINOX [12]. No significant survival difference was observed between patients who received ACT and those who did not [12]. However, in a subgroup analysis, ACT was associated with improved survival in patients with pathology-proven lymph node metastases (26 vs. 13 months), whereas no survival benefit was found in patients with lymph node–negative disease [12]. Similarly, a retrospective study by Sugawara et al. demonstrated that ACT was associated with significantly improved survival in patients with ypT3/ypT4, R1/R2 resections, and moderately/poorly differentiated tumors [13]. Another study reported a survival benefit associated with ACT in patients with tumoral surgical margins at microscopy evaluation [22]. In our study, ACT was associated with improved survival outcomes in patients with a suboptimal response to NACT but not in those who achieved an optimal response, characterized by MPR with pN0/R0, which is consistent with previous findings [12,13,22]. In summary, our findings, along with those from prior studies, suggest that ACT may improve clinical outcome in patients with limited response to NACT including significant residual disease (ypT3/4, ypN1/2, positive surgical margins) but not in patients achieving MPR (TRG-CAP 0–1) with pN0/R0. Further research is warranted to better define the subgroups of residual disease that benefit from ACT.

In our cohort, only 29% of patients achieved MPR, which is consistent with historical data [18,23]. Achieving a TRG-CAP 0–1 was associated with better survival outcomes, as expected, given that TRG is a well-established prognostic factor [18,23]. TRG-CAP became statistically non-significant but showed a trend toward correlation with DFS (*p* = 0.0892) and OS (*p* = 0.1142), most likely due to the limited sample. Although not statistically significant, a trend toward improved OS was observed in patients who received 6 months of perioperative chemotherapy compared with <6 months (*p* = 0.0623). This lack of significance may be due to the limited sample size and underpowered analysis. Another retrospective study found that ACT was associated with improved survival in patients who had received less than 4 months of neoadjuvant therapy [22]. These findings may support ASCO guideline recommendations to complete 6 months of perioperative chemotherapy if feasible [20]. Optimizing ACT regimens based on the pathologic response to NACT is a topic of significant interest in the field; however, few studies have evaluated the correlation between the transition of therapy from NACT to ACT and survival outcomes. Our findings indicate that this transition does not significantly correlate with survival outcomes. Whether to escalate or convert adjuvant therapy and extend the duration of perioperative chemotherapy, particularly in cases of poor response to neoadjuvant treatment such as TRG-CAP 3, is a major area of interest in the field [24]. However, due to the limited sample size, we were not able to perform statistical analyses.

While previous studies have typically defined major pathologic response (MPR) based solely on TRG-CAP [18], our analysis incorporated pN0 and R0 resection into the classification. This composite approach was used given the well-established association between residual nodal disease or positive margins and poorer prognosis [25,26,27]. Biologically, this classification may more comprehensively reflect the extent of pathologic response to neoadjuvant chemotherapy. Nonetheless, further validation of this composite classification—including its prognostic and potential predictive value—should be pursued in external cohorts.

Although our findings suggest that ACT may offer clinical benefit, particularly in patients who do not achieve a major pathologic response, pN0, or R0 resection, whether to give adjuvant chemotherapy, as well as the duration and regimen of perioperative chemotherapy, was determined at the discretion of the treating physician without a pre-specified institutional protocol. Therefore, our results should be considered hypothesis-generating rather than definitive. Prospective validation is warranted before ACT can be safely omitted in patients who achieve a favorable pathologic response following neoadjuvant chemotherapy.

Although perioperative chemotherapy has become a well-established strategy in colorectal cancer with liver metastases [10,27,28,29], offering insights into treatment sequencing and patient selection, its role in resected pancreatic cancer remains less clearly defined. This contrast highlights the need for further refinement of perioperative treatment strategies in pancreatic cancer, potentially informed by lessons learned from colorectal cancer [30].

Our study is not without limitations. First, it was a retrospective, single-center study. There is a risk of overestimating the benefit of ACT due to potential selection bias, as some patients may be ineligible for ACT following curative resection and NACT because of postoperative complications or comorbidities, which can be related to poorer survival outcomes. The study included heterogeneous NACT and ACT regimens, including NACT regimens such as GTX, which were used in the past but have been replaced by more contemporary regimens. This may influence the generalizability of the current study results, although 58% of patients still received contemporary NACT regimens such as mFOLFIRINOX or GEM-NAB. The transition from NACT to ACT was based on the discretion of the treating physician without a pre-specified protocol. Additionally, neoadjuvant radiotherapy (RT) was not controlled; however, the majority of patients (97%) received neoadjuvant RT, making it unlikely to have an impact on the results. Due to the limited sample size, subgroup analyses by NACT regimen (mFOLFIRINOX or GEMNAB) were not performed. Given the exploratory nature of the subgroup analyses and the limited sample sizes within certain subgroups, these findings should be interpreted with caution due to the potential for type I error and limited statistical power. The intolerability to NACT and ACT was not captured in the current analysis. Despite these limitations, our findings have the potential to contribute valuable insights to the field, particularly given the scarcity of data. Moreover, our results are overall consistent with prior studies and may provide an additional layer of evidence to support and enhance previously suggested findings, such as pathological response to NACT being a potential predictive biomarker for ACT in pancreatic cancer patients.

## 5. Conclusions

In conclusion, among PDAC patients undergoing NACT followed by curative resection, achieving TRG-CAP 0–1 and receiving ACT were associated with favorable survival outcomes. Additional ACT appeared to benefit patients who did not achieve an MPR, pN0, or R0 resection to NACT, with limited benefit for those who did MPR with pN0/R0, suggesting that pathological response may serve as a potential predictive marker to guide ACT decisions. In our cohort, the specific NACT regimen (mFOLFIRINOX vs. GEM-NAB) and transitions from NACT to ACT did not significantly impact survival outcomes.

## Figures and Tables

**Table 1:** Patient baseline characteristics and treatment details.

Characteristics		N = 230	% or IQR
Age (years)	Median	68	62–72
Gender	Male	117	51
	Female	113	49
Race	Black	11	5
	White	216	94
	Other	2	1
	Not Available	1	0
NACT Regimen	mFOLFIRINOX	96	42
	GEM-NAB	34	15
	GTX	100	43
Duration of NACT	≤2 months	97	42
	>2 to ≤4 months	25	11
	>4 months	108	47
Neoadjuvant RT	No	6	3
	Yes	224	97
Path T-Stage	0	13	6
	1	59	26
	2	82	36
	3	74	32
	4	2	1
Path N-Stage	0	136	59
	1	81	35
	2	13	6
Surgical Margin Status	R0	216	94
	R1	14	6
Histologic Grade	1	15	7
	2	142	62
	3	54	23
	N/A	19	8
TRG-CAP	0–1	67	29
	2–3	163	71
Receipt of ACT	No	75	33
	Yes	155	67
NACT vs. ACT *	De-escalate	86	56
	Same	39	25
	Conversion	30	19
Duration of Perioperative chemotherapy	<6 months	180	78
	6 months	50	22
Recurred	No	115	50
	Yes	115	50
Died	No	109	47
	Yes	121	52

* Among patients who received ACT (N = 155). Abbreviations: ACT, adjuvant chemotherapy; N/A, not available; NACT, neoadjuvant chemotherapy; path, pathologic; RT, radiation therapy.

## Data Availability

The data presented in this study are available in this article and Supplementary Materials.

## Supplementary Materials

The following supporting information can be downloaded at: https://www.mdpi.com/article/10.3390/cancers17111797/s1, Figure S1: (A) DFS and (B) OS by duration of perioperative chemotherapy; Figure S2. Survival outcomes by neoadjuvant chemotherapy regimen, duration of neoadjuvant chemotherapy, and chemotherapy transition from neoadjuvant to adjuvant therapy (A) DFS and (B) OS by neoadjuvant chemotherapy regimen; (C) DFS and (D) OS by duration of neoadjuvant chemotherapy; and (E) DFS and (F) OS by chemotherapy transition from neoadjuvant to adjuvant therapy.

## References

[1] Cai J., Chen H., Lu M., Zhang Y., Lu B., You L., Zhang T., Dai M., Zhao Y. (2021). Advances in the epidemiology of pancreatic cancer: Trends, risk factors, screening, and prognosis. Cancer Lett..

[2] SEER Cancer of the Pancreas—Cancer Stat Facts. https://seer.cancer.gov/statfacts/html/pancreas.html.

[3] Mizrahi J.D., Surana R., Valle J.W., Shroff R.T. (2020). Pancreatic cancer. Lancet.

[4] Sohal D.P.S., Willingham F.F., Falconi M., Raphael K.L., Crippa S. (2017). Pancreatic Adenocarcinoma: Improving Prevention and Survivorship. Am. Soc. Clin. Oncol. Educ. Book.

[5] Kommalapati A., Tella S.H., Goyal G., Ma W.W., Mahipal A. (2018). Contemporary Management of Localized Resectable Pancreatic Cancer. Cancers.

[6] Strobel O., Neoptolemos J., Jäger D., Büchler M.W. (2019). Optimizing the outcomes of pancreatic cancer surgery. Nat. Rev. Clin. Oncol..

[7] Versteijne E., Vogel J.A., Besselink M.G., Busch O.R.C., Wilmink J.W., Daams J.G., van Eijck C.H.J., Groot Koerkamp B., Rasch C.R.N., van Tienhoven G. (2018). Meta-analysis comparing upfront surgery with neoadjuvant treatment in patients with resectable or borderline resectable pancreatic cancer. Br. J. Surg..

[8] Gillen S., Schuster T., Meyer zum Büschenfelde C., Friess H., Kleeff J. (2010). Preoperative/Neoadjuvant Therapy in Pancreatic Cancer: A Systematic Review and Meta-Analysis of Response and Resection Percentages. PLoS Med..

[9] Versteijne E., Suker M., Groothuis K., Akkermans-Vogelaar J.M., Besselink M.G., Bonsing B.A., Buijsen J., Busch O.R., Creemers G.J., van Dam R.M. (2020). Preoperative Chemoradiotherapy Versus Immediate Surgery for Resectable and Borderline Resectable Pancreatic Cancer: Results of the Dutch Randomized Phase III PREOPANC Trial. J. Clin. Oncol..

[10] Springfeld C., Ferrone C.R., Katz M.H.G., Philip P.A., Hong T.S., Hackert T., Büchler M.W., Neoptolemos J. (2023). Neoadjuvant therapy for pancreatic cancer. Nat. Rev. Clin. Oncol..

[11] NCCN Guidelines Panel (2025). NCCN Clinical Practice Guidelines in Oncology.

[12] van Roessel S., van Veldhuisen E., Klompmaker S., Janssen Q.P., Abu Hilal M., Alseidi A., Balduzzi A., Balzano G., Bassi C., Berrevoet F. (2020). Evaluation of Adjuvant Chemotherapy in Patients With Resected Pancreatic Cancer After Neoadjuvant FOLFIRINOX Treatment. JAMA Oncol..

[13] Sugawara T., Rodriguez Franco S., Sherman S., Kirsch M.J., Colborn K., Ishida J., Grandi S., Al-Musawi M.H., Gleisner A., Schulick R.D. (2023). Association of Adjuvant Chemotherapy in Patients with Resected Pancreatic Adenocarcinoma After Multiagent Neoadjuvant Chemotherapy. JAMA Oncol..

[14] Choi J.H., Kim M.K., Lee S.H., Park J.W., Park N., Cho I.R., Ryu J.K., Kim Y.-T., Jang J.-Y., Kwon W. (2022). Proper adjuvant therapy in patients with borderline resectable and locally advanced pancreatic cancer who had received neoadjuvant FOLFIRINOX. Front. Oncol..

[15] College of American Pathologists (2021). Protocol for the Examination of Specimens from Patients with Carcinoma of the Pancreas.

[16] van Roessel S., Janssen B.V., Soer E.C., Fariña Sarasqueta A., Verbeke C.S., Luchini C., Brosens L.A.A., Verheij J., Besselink M.G. (2021). Scoring of tumour response after neoadjuvant therapy in resected pancreatic cancer: Systematic review. Br. J. Surg..

[17] Amin M.B., Edge S., Greene F., Byrd D.R., Brookland R.K., Washington M.K. (2017). AJCC Cancer Staging Manual.

[18] Bao Q.R., Frigerio I., Tripepi M., Marletta S., Martignoni G., Giardino A., Regi P., Scopelliti F., Allegrini V., Girelli R. (2023). Prognostic value of major pathological response following neoadjuvant therapy for non resectable pancreatic ductal adenocarcinoma. Pancreatology.

[19] Conroy T., Pfeiffer P., Vilgrain V., Lamarca A., Seufferlein T., O’Reilly E.M., Hackert T., Golan T., Prager G., Haustermans K. (2023). Pancreatic cancer: ESMO Clinical Practice Guideline for diagnosis, treatment and follow-up☆. Ann. Oncol..

[20] Khorana A.A., Mangu P.B., Berlin J., Engebretson A., Hong T.S., Maitra A., Mohile S.G., Mumber M., Schulick R., Shapiro M. (2017). Potentially Curable Pancreatic Cancer: American Society of Clinical Oncology Clinical Practice Guideline Update. J. Clin. Oncol..

[21] Groot V.P., Blair A.B., Gemenetzis G., Ding D., Burkhart R.A., Yu J., Borel Rinkes I.H.M., Molenaar I.Q., Cameron J.L., Weiss M.J. (2019). Recurrence after neoadjuvant therapy and resection of borderline resectable and locally advanced pancreatic cancer. Eur. J. Surg. Oncol..

[22] Shimizu T., Maeda S., Link J., Deranteriassian A., Premji A., Verma A., Chervu N., Park J., Girgis M., Benharash P. (2024). Clinical and pathological factors associated with survival in patients with pancreatic cancer who receive adjuvant therapy after neoadjuvant therapy: A retrospective multi-institutional analysis. Surgery.

[23] He J., Blair A.B., Groot V.P., Javed A.A., Burkhart R.A., Gemenetzis G., Hruban R.H., Waters K.M., Poling J., Zheng L. (2018). Is a Pathological Complete Response Following Neoadjuvant Chemoradiation Associated With Prolonged Survival in Patients With Pancreatic Cancer?. Ann. Surg..

[24] Kim S.S., Ko A.H., Nakakura E.K., Wang Z.J., Corvera C.U., Harris H.W., Kirkwood K.S., Hirose R., Tempero M.A., Kim G.E. (2019). Comparison of Tumor Regression Grading of Residual Pancreatic Ductal Adenocarcinoma Following Neoadjuvant Chemotherapy Without Radiation: Would Fewer Tier-Stratification Be Favorable Toward Standardization?. Am. J. Surg. Pathol..

[25] Tummers W.S., Groen J.V., Sibinga Mulder B.G., Farina-Sarasqueta A., Morreau J., Putter H., van de Velde C.J., Vahrmeijer A.L., Bonsing B.A., Mieog J.S. (2019). Impact of resection margin status on recurrence and survival in pancreatic cancer surgery. Br. J. Surg..

[26] Ghaneh P., Kleeff J., Halloran C.M., Raraty M., Jackson R., Melling J., Jones O., Palmer D.H., Cox T.F., Smith C.J. (2019). The Impact of Positive Resection Margins on Survival and Recurrence Following Resection and Adjuvant Chemotherapy for Pancreatic Ductal Adenocarcinoma. Ann. Surg..

[27] Morales-Oyarvide V., Rubinson D.A., Dunne R.F., Kozak M.M., Bui J.L., Yuan C., Qian Z.R., Babic A., Da Silva A., Nowak J.A. (2017). Lymph node metastases in resected pancreatic ductal adenocarcinoma: Predictors of disease recurrence and survival. Br. J. Cancer.

[28] Van Cutsem E., Cervantes A., Adam R., Sobrero A., Van Krieken J.H., Aderka D., Aranda Aguilar E., Bardelli A., Benson A., Bodoky G. (2016). ESMO consensus guidelines for the management of patients with metastatic colorectal cancer. Ann. Oncol..

[29] Yoshino T., Arnold D., Taniguchi H., Pentheroudakis G., Yamazaki K., Xu R.H., Kim T.W., Ismail F., Tan I.B., Yeh K.H. (2018). Pan-Asian adapted ESMO consensus guidelines for the management of patients with metastatic colorectal cancer: A JSMO–ESMO initiative endorsed by CSCO, KACO, MOS, SSO and TOS. Ann. Oncol..

[30] Riesco-Martinez M.C., Modrego A., Espinosa-Olarte P., La Salvia A., Garcia-Carbonero R. (2022). Perioperative Chemotherapy for Liver Metastasis of Colorectal Cancer: Lessons Learned and Future Perspectives. Curr. Treat. Options Oncol..

