# Supplementary material for: Association of Pathologic Response and Adjuvant Chemotherapy with Survival in Resected Pancreatic Ductal Adenocarcinoma Following Neoadjuvant Therapy
Source: Cancers (Basel). 2025 May 28;17(11):1797. doi: 10.3390/cancers17111797 (PMC12153592; doi:10.3390/cancers17111797)
Supplement: Supplementary file 1 [file cancers-17-01797-s001.zip › cancers-3618818-supplementary.pdf]

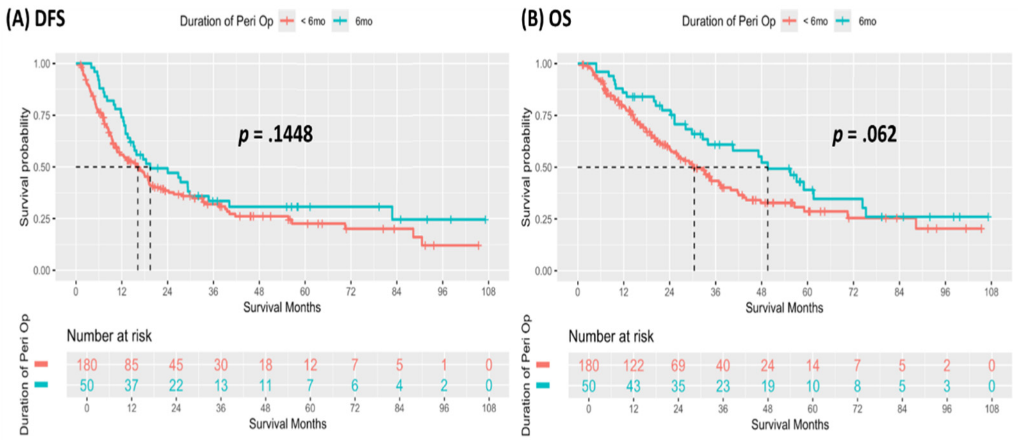

Figure S1. (A) DFS and (B) OS by duration of perioperative chemotherapy.

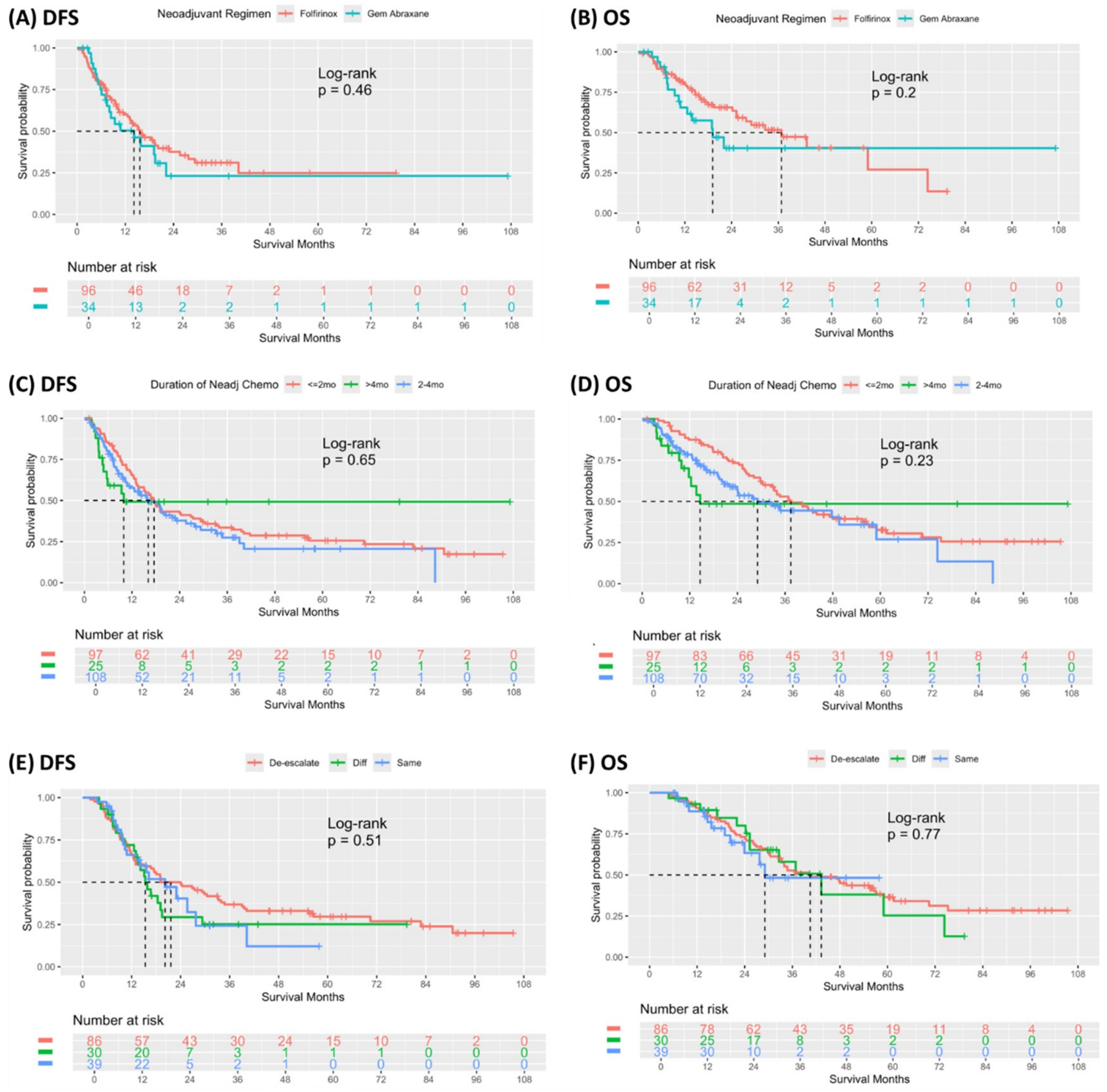

Figure S2. Survival outcomes by neoadjuvant chemotherapy regimen, duration of neoadjuvant chemotherapy, and chemotherapy transition from neoadjuvant to adjuvant therapy (A) DFS and (B) OS by neoadjuvant chemotherapy regimen; (C) DFS and (D) OS by duration of neoadjuvant chemotherapy; and (E) DFS and (F) OS by chemotherapy transition from neoadjuvant to adjuvant therapy.
